# Dietary amino acid intake and sleep duration are additively involved in future cognitive decline in Japanese adults aged 60 years or over: a community-based longitudinal study

**DOI:** 10.1186/s12877-023-04359-2

**Published:** 2023-10-11

**Authors:** Kaori Kinoshita, Rei Otsuka, Michihiro Takada, Yukiko Nishita, Chikako Tange, Hiroko Jinzu, Katsuya Suzuki, Hiroshi Shimokata, Akira Imaizumi, Hidenori Arai

**Affiliations:** 1 Department of Frailty Research, Center for Gerontology and Social Science, Research Institute, National Center for Geriatrics and Gerontology, 7-430 Morioka, Obu, Aichi 474-8511 Japan; 2 Department of Epidemiology of Aging, Center for Gerontology and Social Science, Research Institute, National Center for Geriatrics and Gerontology, Aichi, Japan; 3 Research Institute for Bioscience Products & Fine Chemicals, AJINOMOTO CO., Inc., Kanagawa, Japan; 4 Institute of Food Sciences and Technologies, AJINOMOTO CO., Inc., Kanagawa, Japan; 5 Graduate School of Nutritional Sciences, Nagoya University of Arts and Sciences, Aichi, Japan; 6 National Center for Geriatrics and Gerontology, Aichi, Japan

**Keywords:** Cognitive function, Dietary amino acid, Sleep duration, Prolonged sleep, Older adults

## Abstract

**Background:**

Sleep duration and amino acid intake are independently associated with cognitive decline. This study aimed to determine the longitudinal association between sleep duration and cognitive impairment incidence and to examine the involvement of diet, particularly amino acid intake, in these associations in community dwellers.

**Methods:**

In this longitudinal study in a community-based setting, we analyzed data from 623 adults aged 60–83 years without cognitive impairment at baseline. Sleep duration was assessed using a self-report questionnaire. Amino acid intake was assessed using 3-day dietary records. Cognitive impairment was defined as a Mini-Mental State Examination score ≤ 27. Participants were classified into short-, moderate-, and long-sleep groups according to baseline sleep duration (≤ 6, 7–8, and > 8 h, respectively). Using moderate sleep as a reference, odds ratios (ORs) and 95% confidence intervals (CIs) of short- and long-sleep for cognitive-impairment incidence were estimated using the generalized estimating equation. Participants were classified according to sex-stratified quartiles (Q) of 19 amino acid intake: Q1 and Q2–Q4 were low- and middle to high-intake groups, respectively. Using middle- to high-intake as a reference, ORs and 95% CIs of low intake for cognitive impairment incidence were estimated using the generalized estimating equation in each sleep-duration group. Follow-up period, sex, age, body mass index, depressive symptoms, education, smoking status, employment status, sleep aids use, physical activity, medical history, and Mini-Mental State Examination score at baseline were covariates.

**Results:**

Mean follow-up period was 6.9 ± 2.1 years. Adjusted ORs (95% CIs) for cognitive impairment in short- and long-sleep groups were 0.81 (0.49–1.35, P = 0.423) and 1.41 (1.05–1.87, P = 0.020), respectively. Particularly in long sleepers (i.e., > 8 h), cognitive impairment was significantly associated with low cystine, proline, and serine intake [adjusted ORs (95% CIs) for cognitive impairment were 2.17 (1.15–4.11, P = 0.017), 1.86 (1.07–3.23, P = 0.027), and 2.21 (1.14–4.29, P = 0.019), respectively].

**Conclusions:**

Community-dwelling adults aged ≥ 60 years who sleep longer are more likely to have cognitive decline, and attention should be paid to the low cystine, proline, and serine intake.

**Supplementary Information:**

The online version contains supplementary material available at 10.1186/s12877-023-04359-2.

## Background

The number of people affected by dementia is increasing with the rapid growth of the older population worldwide, and it is estimated that people with dementia will account for 5–8% of those 60 years and older in 2050 [1]. Therefore, slowing down cognitive decline by targeting modifiable factors related to brain function is important. To this end, it is necessary to consider lifestyle factors such as sleep and diet [2, 3].

A U-shaped association between sleep duration and cognitive impairment has been reported in systematic reviews and meta-analyses [4–7]. Sleep deprivation has been suggested to cause cognitive disorders owing to amyloid-beta accumulation in the brain; however, the mechanism by which prolonged sleep duration leads to cognitive decline has not yet been elucidated [8]. Several studies on the association between sleep and nutrients have been reported. Long sleepers have been reported to have lower protein intake than normal sleepers [9, 10]. A recently reported systematic review and meta-analysis revealed that high protein intake may help to improve sleep conditions [11]. Tryptophan is involved in the synthesis of serotonin and melatonin, which play a role in sleep-wake cycle, and glutamine is used to synthesize gamma-aminobutyric acid, which promotes sleep induction. Additionally, studies have found an association between low protein intake and cognitive impairment [12, 13], and the negative association between protein intake and cognitive function has been attributed to the involvement of specific amino acids [14, 15].

Several studies have suggested a possible link between amino acids, sleep, and cognitive function. In long sleepers, inflammatory cytokines are increased [16]. Inflammation is one of the causes of neurological diseases [17] and enhances oxidative stress, which is also considered an important factor in cognitive decline [18–20]. Several amino acids that comprise dietary proteins inhibit inflammation [21] and reduce oxidative stress [22]. In addition, some amino acids may play a role in cognitive function, including those acting as neurotransmitters and those used for the synthesis of neurotransmitters. However, to our knowledge, no previous studies have examined the association between amino acids, sleep, and cognitive function.

We hypothesized amino acid intake to be one of the underlying factors in the association between long sleep duration and cognitive decline. Long sleepers have lower protein intake than normal sleepers and low protein intake is associated with cognitive decline; furthermore, amino acids, which comprise proteins, play a role in cognitive function [14, 15]. Based on these considerations, it is reasonable to focus on low rather than high intake of amino acids and investigate their association with future cognitive decline. Here, we aimed to: (1) to determine the longitudinal association between sleep duration and the incidence of cognitive impairment and (2) examine whether diet, particularly amino acid intake, was involved in these associations in community-dwelling adults aged 60 years and older.

## Methods

### Study design and participants

This study was derived from the National Institute for Longevity Sciences-Longitudinal Study of Aging (NILS-LSA), and its design has been previously described [23]. The NILS-LSA participants were recruited using stratified random sampling by age and sex among community dwellers from Obu City and Higashiura Town in Aichi Prefecture, Japan. Surveys have been conducted every 2 years since 1997 (i.e., for each survey, all participant examinations were completed over 2 years). Participants were aged 40–79 years at their first participation.

In the present study, the third survey of the NILS-LSA (between May 2002 and May 2004; n = 2378) was set as the baseline because of the British bovine spongiform encephalopathy epidemic that also affected Japan during the second NILS-LSA survey (between April 2000 and May 2002) [24]. The bovine spongiform encephalopathy outbreak led to decreased meat consumption, which is the main source of amino acids. Because the NILS-LSA assesses the Mini-Mental State Examination (MMSE) score only for participants aged 60 years and older, this age group was included in the present study. At baseline, there were 1202 participants aged ≥ 60 years; 1005 participated in the follow-up survey (4th to 7th survey, up to July 2012) at least once. Of them, the following were excluded: patients with no cognitive function assessment (n = 10), no dietary records (n = 49), cognitive impairment at baseline (as defined by MMSE score ≤ 27, n = 309), and missing covariate data (n = 14). Finally, data from 623 participants were analyzed in this study (age range, 60–83 years; male participants, n = 296, 47.5%).

This study was conducted in accordance with the Declaration of Helsinki, and all procedures were approved by the Ethics Committee of Human Research of the National Center for Geriatrics and Gerontology, Japan (No. 1447-2) and the Ethics Committee of Ajinomoto Co., Inc. (No. 2020-007). Written informed consent was obtained from all participants.

### Assessment of cognitive function

Cognitive function was assessed using the Japanese version of the MMSE (range 0–30, with higher scores indicating better cognitive function) [25, 26] by a trained psychologist or psychology graduate students in both baseline and follow-up surveys. A recent study that examined the validity and reliability of the Japanese version of MMSE reported that an MMSE score ≤ 27 is optimal for detecting mild cognitive impairment in Japanese adults [27]. In line with previous studies suggesting the same cut-off values, the present study defined MMSE score of ≤ 27 as cognitive impairment [27–30]. The number of participants with MMSE scores ≤ 23, which is generally used as a cut-off for suspected dementia, was too small to be analyzed in the present study [25, 26].

### Assessment of sleep duration

Sleep duration was assessed using a questionnaire for intensity and frequency of activity during the year, which is a semi-quantitative assessment tool for calculating the metabolic equivalents (METs) score [31]. The questionnaire included detailed monthly records of leisure time and physical activity at work over the previous 12 months. In addition, daily sleep durations (i.e., the hours of sleep per day) were recorded, excluding naps, every 2 months. The average sleep duration calculated from the previous 12 months’ sleep duration records was used in this study as habitual sleep duration. Trained staff members performed the assessments.

### Assessment of dietary intake

Baseline average dietary intake was assessed using a 3-day dietary record, which included the intake data from two weekdays and one weekend day [32]. Reproducibility in 3-day dietary surveys has been demonstrated in a study of adults and children [33]. Another study has shown similar levels of reliability for 3-day and 6-day dietary records [34]. Furthermore, in our study, efforts were made to improve the accuracy of the dietary record by capturing pre- and post-meal photographs in addition to the food records and having the recording forms checked by a skilled dietitian. Specifically, each food item was weighed using a kitchen scale before or after cooking (Sekisui Jushi, Tokyo, Japan), and photos of the meals were captured before and after eating, using a disposable camera (Fuji Film, Tokyo, Japan). Dietitians used these photos to help complete food consumption estimates and telephoned participants to resolve any discrepancies or obtain further information when necessary. The average dietary intake for each meal was calculated based on the Standard Tables of Food Composition in Japan [35].

### Other measurements

From anthropometric data, the body mass index (BMI) was calculated using the following formula: BMI = weight (kg)/height² (m²). The following items were assessed using self-reported questionnaires: medical history (hypertension, ischemic heart disease, dyslipidemia, diabetes mellitus, and stroke), years of education, smoking status, employment status, and medication use (hypnotics, sedatives, or anxiolytics). Depressive symptoms were assessed using the self-reported Center for Epidemiologic Studies Depression Scale (CES-D, range 0–60, with higher scores indicating more severe depressive symptoms) [36]. Daily total physical activity (MET-min/d) was assessed by a trained staff member using the questionnaire for intensity and frequency of activity during the year [31]. These variables at baseline were used as covariates.

### Statistical analyses

All statistical analyses were performed using Statistical Analysis System version 9.3 (SAS Institute, Cary, NC, USA). A two-sided P value of < 0.05 was considered statistically significant. Continuous variables are presented as mean ± standard deviation (SD), and categorical variables are presented as numbers and percentages (%).

Statistical analysis was conducted to determine (1) the association between sleep duration and the incidence of cognitive impairment and (2) the association between amino acid intake and the incidence of cognitive impairment stratified by sleep duration.

First, participants were classified into short-, moderate-, and long-sleep groups according to their baseline sleep duration (≤ 6 h, 7–8 h, and > 8 h, respectively), following the results of a systematic review [7]. Comparisons of the baseline characteristics of study participants according to the short-, moderate-, and long-sleep groups were analyzed using the chi-square test or Fisher’s exact test for categorical values and the general linear model for continuous variables. The longitudinal association between sleep duration and the incidence of cognitive impairment was analyzed using the generalized estimating equation (GEE) [37]. The GEE analysis was performed using the GENMOD procedure in SAS. Using the moderate-sleep group as a reference, the GEE was applied to estimate the odds ratios (ORs) and 95% confidence intervals (CIs) of the short- and long-sleep groups for the incidence of cognitive impairment. We adjusted for the follow-up period, sex, age (years), BMI (kg/m²), CES-D (score), education (0–7 years/ 8–15 years/ ≥16 years), smoking status (current/ no), employment status (yes/no), use of hypnotics, sedatives, or anxiolytics (yes/no), physical activity (MET-min/d), and MMSE (score) at baseline in Model 1. In Model 2, a history of stroke, hypertension, ischemic heart disease, dyslipidemia, and diabetes mellitus were added to the variables in Model 1.

Second, participants were classified according to sex-stratified quartiles (Q1 to Q4) of 19 amino acid intake. As in a previous study [38], groups Q1 and Q2–Q4 were defined as the low- and middle to high-intake groups, respectively. To perform analyses stratified by sleep duration, participants were reclassified as short to moderate sleepers (≤ 8 h) and long-sleepers (> 8 h) because the number of participants in the short-sleep group was small (n = 58) and a significant association with the incidence of cognitive impairment was only observed in the long-sleep group. The GEE was used to analyze the association between amino acid intake and incident cognitive impairment in short to moderate sleepers (≤ 8 h) and long-sleepers (> 8 h). Using the middle to high-intake group as a reference, the ORs and 95% CIs of the low-intake group for the incidence of cognitive impairment was estimated after an adjustment for the follow-up period, sex, age (year), BMI (kg/m²), CES-D (score), education (0–7 years/ 8–15 years/ ≥16 years), smoking status (current or no), employment status (yes/no), use of hypnotics, sedatives, or anxiolytics (yes/no), physical activity (MET-min/d), MMSE (score), and a history of stroke, hypertension, ischemic heart disease, dyslipidemia, and diabetes mellitus at baseline in Model 1. In Model 2, energy intake (kcal/day) was added to the variables in Model 1. In Model 3, protein intake (g/day) was added to the variables in Model 1. To verify this association, an additional analysis was performed in the same models (i.e., model 1 to model 3) using GEE. In the additional analysis, participants were classified into four groups based on amino acid intake (i.e., low- and middle to high-intake) and sleep duration (i.e., ≤ 8 h and > 8 h): low-intake with > 8 h sleep; low-intake with ≤ 8 h sleep; middle to high-intake with > 8 h sleep; and middle to high-intake with ≤ 8 h sleep. Using the middle to high-intake with ≤ 8 h sleep group as the reference, the ORs and 95% CIs for cognitive impairment were estimated.

In the first supplementary analysis, comparisons of food consumption (g/100 kcal/day) between the low- and the middle to high-intake groups of cystine, proline, and serine were analyzed using the t-test. Cystine, proline, and serine were selected based on the significant association between amino acid intake and the incidence of cognitive impairment calculated by the GEE.

In the second supplementary analysis, to examine the dose-response relationship between amino acid intake and cognitive function, the ORs and 95% CIs for the incidence of cognitive impairment in Q2 to Q4 amino acid intake groups were estimated in long sleepers, using the Q1 amino acid intake group as a reference.

In the third supplementary analysis, the multivariable-adjusted association between the ratio of amino acids to protein intake and cognitive function in long-sleepers was analyzed using GEE to interpret the study results from multiple perspectives. The ratio of each amino acid to total daily protein intake was calculated, and ORs and 95% CIs for incidence of cognitive impairment were estimated.

## Results

Table 1 shows the number of participants in each survey from baseline to follow-up. The minimum and maximum follow-up periods were 1.9 and 8.9 years, respectively. Table 2 shows the baseline characteristics of the study participants according to their sleep duration. The longer the participants slept, the higher their age and the lower their physical activity (P < 0.05, for both group differences and trends).

**Table 1:** Number of participants in each survey from baseline until follow-up

			Cognitive impairment^*^	
	Total n	%	n^†^	%^‡^
Third survey (baseline)	623	100	0	0
Fourth survey	610	97.9	146	23.9
Fifth survey	549	88.1	149	27.1
Sixth survey	480	77.0	135	28.1
Seventh survey	429	68.9	133	31.0
Cumulative number of participants	2691		563	
	Mean	SD		
Follow-up period (y)	6.9	2.1		
Number of follow-up survey participation (range: 1–4)	3.4	1.0		

^*^Assessed using an MMSE score of ≤ 27

^†^Number of incidences of cognitive impairment in each survey

^‡^Percentage of the incidence of cognitive impairment in each survey

SD, standard deviation; MMSE, Mini-Mental State Examination

**Table 2:** Baseline characteristics of participants

	Group of sleep duration^*^				
	Short sleep	Moderate sleep	Long sleep	P value	
	(n = 58)	(n = 388)	(n = 177)	Diff.	Trend
Sleep duration, h	5.7 ± 0.5	7.4 ± 0.5	9.0 ± 0.6		
Male participants	23 (39.7)	175 (45.1)	98 (55.4)	0.033	
Age	68.3 ± 5.6	67.7 ± 5.6	70.1 ± 5.8	< 0.001	0.035
60–69 years	34 (58.6)	255 (65.7)	90 (50.8)	0.004	
70–79 years	23 (39.7)	126 (32.5)	76 (42.9)		
≥80 years	1 (1.7)	8 (2.1)	11 (6.2)		
BMI, kg/m²	23.4 ± 2.9	23.1 ± 2.9	22.7 ± 2.7	0.140	0.108
CES-D, range: 0–60	8.6 ± 8.3	6.5 ± 6.5	8.0 ± 6.8	0.010	0.571
MMSE, range: 0–30	29.0 ± 0.7	29.0 ± 0.8	28.9 ± 0.8	0.543	0.297
Education					
0–7 years	1 (1.7)	1 (0.3)	4 (2.3)	0.143^†^	
8–15 years	52 (89.7)	342 (88.1)	155 (87.6)		
≥ 16 years	5 (8.6)	46 (11.9)	18 (10.2)		
Current smoker	3 (5.2)	48 (12.3)	32 (18.1)	0.028	
Employment	15 (25.9)	147 (37.8)	48 (27.1)	0.019	
Physical activity, MET-min/d	1961.9 ± 133.4	1921.7 ± 195.6	1814.8 ± 130.8	< 0.001	< 0.001
Using of hypnotics/sedatives/anxiolytics^‡^	2 (3.4)	15 (3.9)	8 (4.5)	0.909	
Stroke	3 (5.2)	20 (5.2)	9 (5.1)	0.999	
Hypertension	26 (44.8)	138 (35.6)	78 (44.1)	0.087	
Ischemic heart disease	8 (13.8)	28 (7.2)	23 (13.0)	0.048	
Dyslipidemia	17 (29.3)	105 (27.1)	35 (19.8)	0.138	
Diabetes mellitus	6 (10.3)	39 (10.1)	18 (10.2)	0.997	
Nutritional intake					
Energy, kcal/d	1987.2 ± 390.0	2046.5 ± 400.2	2033.7 ± 399.4	0.567	0.441
Protein, g/d	78.2 ± 17.4	80.9 ± 17.2	77.1 ± 16.0	0.041	0.671
Fat, g/d	52.9 ± 14.1	53.8 ± 13.7	50.4 ± 14.8	0.031	0.239
Carbohydrate, g/d	283.5 ± 53.9	293.4 ± 64.8	293.4 ± 56.1	0.504	0.289

^*^Sleep duration is calculated over 24 h. Short sleep was defined as sleep duration ≤ 6 h, moderate sleep was defined as sleep duration of 7–8 h, and long sleep was defined as sleep duration > 8 h

^†^Obtained using Fisher’s exact test

^‡^Categorized based on the therapeutic category of drugs in Japan (No.112)

Data are presented as the mean ± SD or n (%). P-values were obtained using the chi-square test or Fisher’s exact test for categorical variables, and the general linear model for continuous variables

BMI, body mass index; CES-D, Center for Epidemiologic Studies Depression Scale; MMSE, Mini-Mental State Examination; MET, metabolic equivalents; SD, standard deviation

Table 3 shows the multivariable-adjusted association between sleep duration and the incidence of cognitive impairment. In all models, the adjusted OR for the incidence of cognitive impairment was significantly higher in the long-sleep group than in the moderate-sleep group (P < 0.05), but no significant association was observed in the short-sleep group.

**Table 3:** Multivariable-adjusted association between sleep duration and incidence of cognitive impairment

	Crude			Model 1			Model 2		
Group of sleep duration^*^	OR	95%CI	P value	OR	95%CI	P value	OR	95%CI	P value
Short sleep	0.82	0.50–1.35	0.427	0.79	0.48–1.32	0.376	0.81	0.49–1.35	0.423
Moderate sleep	Ref.			Ref.			Ref.		
Long sleep	1.58	1.22–2.06	0.001	1.41	1.07–1.87	0.016	1.41	1.05–1.87	0.020

^*^Sleep duration is calculated over 24 h. Short sleep was defined as sleep duration ≤ 6 h, moderate sleep was defined as sleep duration of 7–8 h, and long sleep was defined as sleep duration > 8 h

ORs and 95% CIs were estimated using the generalized estimating equations

Model 1: adjusted for sex, age (60–69 y/70–79 y/≥80 y), BMI (kg/m²), MMSE (score), CES-D (score), education (0–7 y/8–15 y/≥16 y), smoking status (current/not), employment status (yes/no), use of hypnotics, sedatives, or anxiolytics (yes/no), physical activity (MET-min/d) at baseline, and follow-up period (y)

Model 2: adjusted for history of stroke, hypertension, ischemic heart disease, dyslipidemia, and diabetes mellitus, in addition to the variables in Model 1

OR, odds ratio; CI, confidence interval; Ref., reference; BMI, body mass index; MMSE, Mini-Mental State Examination; CES-D, Center for Epidemiologic Studies Depression Scale; MET, metabolic equivalents

Table 4 shows the multivariable-adjusted association between amino acid intake and the incidence of cognitive impairment in the long-sleep group (> 8 h). Using the middle to high-intake groups as a reference, significant associations were found between the groups with a low intake of cystine (i.e., ≤ 1038.6 mg/day for males and ≤ 864.0 mg/day for females), proline (i.e., ≤ 3739.4 mg/day for males and ≤ 3295.1 mg/day for females), and serine (i.e., ≤ 3207.4 mg/day for males and ≤ 2685.9 mg/day for females) and the incidence of cognitive impairment. Additionally, the analysis of four groups that combined amino acid (cystine, proline, and serine) intake (i.e., low- and middle to high-intake) and sleep duration (i.e., ≤ 8 h and > 8 h) showed a significant positive association with cognitive impairment only in the low-intake with > 8 h sleep group [OR (95% CI): cystine, 2.15 (1.29–3.57), P = 0.003; proline, 1.97 (1.25–3.13), P = 0.004; serine, 2.33 (1.42–3.81), P = 0.001] (Supplemental Table 1).

**Table 4:** Multivariable-adjusted longitudinal association between amino acid intake and incidence of cognitive impairment

	Crude^*^			Model 1^*^			Model 2^*^			Model 3^*^		
Amino acids	OR	95%CI	P value	OR	95%CI	P value	OR	95%CI	P value	OR	95%CI	P value
Long-sleep group (> 8 h, n = 177)												
Isoleucine	1.51	0.98–2.32	0.060	1.35	0.85–2.15	0.209	1.39	0.80–2.44	0.246	1.72	0.90–3.26	0.099
Leucine	1.52	1.00–2.33	0.053	1.37	0.85–2.19	0.195	1.42	0.81–2.49	0.226	1.75	0.92–3.35	0.089
Lysine	1.38	0.89–2.12	0.149	1.18	0.74–1.87	0.487	1.16	0.69–1.93	0.581	1.33	0.70–2.52	0.389
Methionine	1.39	0.91–2.13	0.132	1.21	0.75–1.95	0.437	1.20	0.68–2.11	0.532	1.37	0.72–2.59	0.335
Cystine	1.76	1.13–2.73	0.012	1.51	0.95–2.41	0.084	1.67	0.96–2.90	0.072	2.17	1.15–4.11	0.017
Phenylalanine	1.60	1.03–2.47	0.035	1.40	0.86–2.27	0.171	1.48	0.83–2.63	0.188	1.88	0.96–3.68	0.064
Tyrosine	1.51	0.98–2.32	0.060	1.30	0.82–2.08	0.269	1.33	0.76–2.32	0.324	1.63	0.84–3.13	0.146
Threonine	1.40	0.91–2.17	0.125	1.24	0.78–1.98	0.364	1.24	0.72–2.14	0.437	1.47	0.78–2.78	0.236
Tryptophan	1.53	0.99–2.37	0.057	1.28	0.80–2.06	0.302	1.30	0.75–2.26	0.354	1.55	0.81–2.96	0.185
Valine	1.55	1.01–2.38	0.043	1.38	0.86–2.22	0.179	1.44	0.82–2.54	0.207	1.79	0.94–3.44	0.078
Histidine	1.25	0.80–1.96	0.321	1.03	0.62–1.71	0.912	0.98	0.57–1.71	0.957	1.04	0.53–2.02	0.919
Arginine	1.55	1.00–2.41	0.050	1.36	0.85–2.17	0.201	1.39	0.82–2.34	0.220	1.69	0.91–3.16	0.099
Alanine	1.48	0.96–2.27	0.074	1.21	0.77–1.93	0.410	1.21	0.70–2.08	0.496	1.41	0.72–2.75	0.316
Aspartic acid	1.63	1.05–2.52	0.028	1.35	0.84–2.16	0.213	1.38	0.80–2.36	0.244	1.69	0.88–3.26	0.117
Glutamic acid	1.29	0.82–2.01	0.266	1.16	0.73–1.84	0.538	1.13	0.65–1.97	0.667	1.28	0.69–2.36	0.429
Glycine	1.43	0.92–2.24	0.112	1.12	0.70–1.78	0.639	1.08	0.63–1.86	0.780	1.22	0.64–2.33	0.547
Proline	1.61	1.05–2.49	0.031	1.52	0.96–2.39	0.072	1.58	0.94–2.63	0.082	1.86	1.07–3.23	0.027
Serine	1.80	1.17–2.77	0.008	1.52	0.95–2.45	0.081	1.68	0.93–3.01	0.084	2.21	1.14–4.29	0.019
Hydroxyproline	1.11	0.72–1.71	0.639	0.92	0.55–1.53	0.737	0.89	0.53–1.52	0.681	0.90	0.50–1.60	0.712
Short to moderate-sleep group (6–8 h, n = 446)												
Isoleucine	1.35	0.94–1.95	0.109	1.28	0.86–1.91	0.226	1.41	0.89–2.24	0.141	1.46	0.89–2.37	0.131
Leucine	1.37	0.95–1.97	0.096	1.33	0.89–1.98	0.163	1.50	0.94–2.38	0.088	1.57	0.96–2.57	0.074
Lysine	1.31	0.91–1.88	0.149	1.28	0.85–1.91	0.236	1.37	0.88–2.14	0.163	1.43	0.88–2.34	0.153
Methionine	1.09	0.75–1.59	0.661	1.09	0.72–1.65	0.687	1.13	0.72–1.78	0.594	1.09	0.66–1.80	0.739
Cystine	1.23	0.85–1.79	0.268	1.13	0.76–1.69	0.554	1.20	0.75–1.91	0.446	1.15	0.71–1.89	0.568
Phenylalanine	1.41	0.98–2.03	0.067	1.34	0.90–1.99	0.157	1.52	0.95–2.41	0.080	1.59	0.97–2.61	0.069
Tyrosine	1.32	0.91–1.92	0.137	1.25	0.84–1.86	0.277	1.38	0.87–2.21	0.174	1.41	0.86–2.32	0.178
Threonine	1.29	0.89–1.87	0.171	1.24	0.83–1.85	0.293	1.35	0.86–2.14	0.196	1.39	0.84–2.28	0.199
Tryptophan	1.40	0.97–2.01	0.071	1.33	0.89–1.97	0.164	1.48	0.94–2.33	0.090	1.55	0.95–2.53	0.077
Valine	1.32	0.91–1.91	0.147	1.27	0.84–1.90	0.255	1.41	0.88–2.26	0.158	1.44	0.87–2.37	0.156
Histidine	1.13	0.78–1.64	0.508	1.12	0.75–1.68	0.582	1.17	0.75–1.84	0.492	1.14	0.70–1.86	0.601
Arginine	1.22	0.84–1.78	0.294	1.18	0.80–1.76	0.406	1.27	0.81–1.98	0.296	1.26	0.78–2.02	0.340
Alanine	1.40	0.97–2.03	0.075	1.36	0.91–2.02	0.134	1.49	0.96–2.32	0.076	1.59	0.99–2.57	0.056
Aspartic acid	1.25	0.87–1.81	0.225	1.15	0.77–1.72	0.491	1.22	0.77–1.92	0.391	1.20	0.73–1.97	0.464
Glutamic acid	1.14	0.78–1.65	0.499	1.02	0.67–1.54	0.935	1.05	0.65–1.69	0.856	0.98	0.60–1.58	0.925
Glycine	1.31	0.90–1.89	0.159	1.28	0.87–1.90	0.215	1.41	0.90–2.20	0.137	1.44	0.90–2.31	0.129
Proline	1.25	0.87–1.79	0.234	1.07	0.72–1.60	0.732	1.12	0.71–1.77	0.635	1.06	0.68–1.66	0.796
Serine	1.27	0.88–1.84	0.196	1.22	0.82–1.82	0.329	1.33	0.84–2.12	0.227	1.33	0.81–2.19	0.261
Hydroxyproline	1.21	0.85–1.75	0.293	1.34	0.90–1.99	0.146	1.38	0.92–2.07	0.122	1.39	0.90–2.14	0.137

^*^ORs and 95% CIs of the low-intake groups of each amino acid intakes were shown, using the middle to high-intake groups of them as the references that performed using the generalized estimating equations

Model 1: adjusted for sex, age (60–69 y/70–79 y/≥80 y), BMI (kg/m²), MMSE (score), CES-D (score), education (0–7 y/8–15 y/≥16 y), smoking status (current/not), employment status (yes/no), using of hypnotics, sedatives, or anxiolytics (yes/no), physical activity (MET-min/d), history of stroke, hypertension, ischemic heart disease, dyslipidemia, and diabetes mellitus at baseline, and follow-up period (y)

Model 2: adjusted for energy intake (kcal/d) in addition to the variables in model 1

Model 3: adjusted for protein intake (g/d) in addition to the variables in model 1

OR, odds ratio; CI, confidence interval; BMI, body mass index; MMSE, Mini-Mental State Examination; CES-D, The Center for Epidemiologic Studies Depression Scale; MET, metabolic equivalents

In the short to moderate-sleep group (≤ 8 h), no significant associations were observed between the incidence of cognitive impairment and amino acid intake. Supplemental Table 2 shows participant food consumption (g/100 kcal/day) according to the low- and middle to high-intake groups of cystine, proline, and serine in the long-sleep group. Cereal grain consumption was significantly higher in the low-intake groups of cystine, proline, and serine than in the middle to high-intake groups (P < 0.01 all). Bean and legume consumption was significantly lower in the low-intake groups of cystine and serine than in the middle to high-intake groups (P < 0.01). Vegetable consumption and fish and seafood consumption were significantly lower in the low-intake groups of serine than in the middle to high-intake groups (P = 0.007 for vegetables, P = 0.010 for fish and seafood). Meat and egg consumption was significantly lower in the low-intake groups of cystine than in the middle to high-intake groups (P = 0.029 for meat and P = 0.004 for eggs). Milk and dairy products were significantly lower in the low-intake proline groups than in the middle to high-intake groups (P < 0.001).

In the second supplementary analysis, the association between the Q1 to Q4 amino acid intake groups and cognitive function showed that the ORs for the incidence of cognitive impairment tended to be lower in groups Q2 to Q4 than in groups Q1, but no statistically significant dose-response relationship was found (Supplemental Table 3).

In the third supplementary analysis, the association between the ratio of amino acids to protein intake and cognitive function, are presented in Supplemental Table 4. No significant association was found.

## Discussion

The present study found that a long sleep duration (> 8 h) was significantly associated with the incidence of cognitive impairment among community dwellers aged 60 years and older, with a maximum follow-up period of 8.9 years. Furthermore, among long sleepers, those with a low intake of cystine, proline, and serine had significantly higher ORs for cognitive impairment than those with a middle to high intake of these amino acids. Additional analysis that performed to confirm this association (Supplemental Table 1) shows that the ORs of these three amino acids are significantly higher only in the ‘’low intake with long sleep’’ group, suggesting that especially long sleepers should be aware of low intakes of these amino acids. To our knowledge, this is the first study to focus on the relationship between amino acid intake and cognitive function considering sleep duration. Our findings suggest that adults aged 60 years and older who sleep longer are more likely to have cognitive decline, and attention should be paid to the poor intake of cystine, proline, and serine. These results may contribute to the development of nutritional approaches to maintain good brain function in older adults.

Although a few studies have reported no significant association between long sleep duration and cognitive function decline [39–41], our results are consistent with many other studies that have reported the association of long sleep duration with cognitive impairment [42–47]. Significant associations between short sleep duration and cognitive impairment have also been reported [4, 5, 7, 48, 49], although no significant associations were observed in the present study. This may be owing to the small number of participants with short sleep duration in this study (n = 58). Additionally, recent studies have reported that a sleep duration of 4 h or less is associated with cognitive decline or development of dementia, while sleep durations of 5–6 h are not associated with it [4, 50]. The mean sleep duration of the short-sleep group in the present study was 5.7 h, and only one participant had ≤ 4 h sleep duration.

Although no significant dose-response relationship was found between amino acid intake and cognitive function, the low intake of cystine, proline, and serine was significantly associated with cognitive impairment in the long sleeper group. Therefore, it may be important to pay attention to dietary habits to avoid inadequate intake of these amino acids. The possibility that low intake of cystine, proline, and serine may lead to cognitive decline in long sleepers can be discussed based on the results of several previous studies. Meta-analyses have shown an increase in inflammatory cytokines in long-sleepers [16]. Inflammation is known to be one of the causes of neurological diseases, such as Alzheimer’s disease [17], and to enhance oxidative stress, which is also considered an important factor in cognitive decline [18–20]. Cystine intake reduces inflammation [21] and oxidative stress because intestinal bacteria produce antioxidants from cystine [22]. Proline-containing peptide intake improves cognitive function, including short- and long-term memory, by suppressing the production of inflammatory cytokines and oxidative stress [51–53]. Although serine is a non-essential amino acid synthesized in the body, it is essential for the normal growth of neurons [54], and its intake suppresses both synaptic and behavioral deficits in the mouse models of Alzheimer’s disease [55]. Serine is important for synaptic transmission, as it is involved in the biosynthesis of sphingolipids and phospholipids, which are abundant in the brain. The serum serine level decreased with age in both males and females [56], which emphasizes the significance of dietary serine intake in older adults to ensure an adequate amount of serine for the maintenance of cognitive function.

In the comparison of food consumption between the low- and the middle to high-intake groups of cystine, proline, and serine in long sleepers (i.e., sleep duration > 8 h) in the present study (Supplemental Table 2), the low-intake groups had significantly lower consumption of beans and legumes, vegetables, fish and seafood, meat, eggs, and milk and dairy products, and significantly higher consumption of cereal grains than the middle to high-intake groups. A previous study on the Japanese population reported that dietary patterns with a low intake of rice and a high intake of beans and legumes, vegetables, and milk and dairy products were associated with a reduced risk of dementia [57]. In contrast, in short to moderate sleepers (i.e., sleep duration ≤ 8 h) in the present study, the ORs of the low intake of leucine, phenylalanine, tryptophan, and alanine for the incidence of cognitive impairment were approximately 1.6, which was high, although not significant. These amino acids have been suggested to play a role in cognitive function because of their involvement in neurotransmission and as an energy source for the brain [14, 15]. Phenylalanine and alanine were associated with future cognitive decline in a previous study analyzing the same cohort without taking sleep duration into account [38], which was reaffirmed in the present analysis. Therefore, a diet rich in amino acids may be important for maintaining cognitive function regardless of sleep duration. By contrast, the present research suggests that people who sleep long hours should be particularly careful not to under-consume certain characteristic amino acids. For long sleepers, it may be important to pay attention to cystine, proline, and serine insufficiencies by incorporating diets with fewer cereal grains and more beans and legumes, vegetables, fish and seafood, meat, eggs, milk, and dairy products.

The present study had some limitations. First, because we focused on amino acid intake, we did not consider the influence of nutritional balance and other nutrients. Multiple nutrients are consumed through the diet, and the function of nutrients other than amino acids or interactions between nutrients may have affected cognitive function. Typically, fish consumption has been reported to reduce the risk of dementia by a systematic review and a dose-response meta-analysis [58]. Fish are rich in amino acids, but are also rich in eicosapentaenoic and docosahexaenoic fatty acids, which are well-known as preventive factors of cognitive decline. Additionally, ingredients such as phytochemicals or antioxidants that could not be estimated in the Japanese Food Composition Tables may have affected cognitive function [59, 60]. Second, amino acid intake was assessed only at baseline. As dietary habits are influenced by factors such as the chewing function, decreased income, and limited food access that older adults are more likely to face, nutritional intake may change with aging [61]. Third, although sleep duration was assessed by trained staff members, hours of sleep data were based on self-reported records without objective measurements, such as the ActiGraph. However, self-reported sleep duration has been reported to be moderately correlated with that measured by ActiGraph and health-related factors [62, 63]; thus, self-reported sleep duration is considered to be close to an objective measurement. Finally, in order to interpret the results from multiple perspectives, the study also analyzed the association between the ratio of amino acids to protein intake and cognitive function, but found no significant association. This may be because the present study focused on total daily intake of amino acids, and therefore did not examine each meal separately. The ratio of amino acids to proteins affects the bioavailability of dietary proteins [64]. However, since digestion and absorption of amino acids occur after meals, the ratio of amino acids to proteins should be considered on a meal-by-meal basis. It is also important that future studies examine whether amino acid intake at breakfast, lunch, and dinner affects cognitive function.

## Conclusions

Long sleep duration was significantly associated with the incidence of cognitive impairment during the follow-up period (maximum 8.9 years) compared with moderate sleep duration in Japanese community-dwelling adults 60 years and older. In participants with long sleep duration, the low intake of cystine, proline, and serine predicted cognitive impairment incidence, which may be involved in maintaining cognitive function by suppressing inflammation and oxidative stress or improving synaptic transmission. Our findings may provide insights into nutritional approaches for maintaining good brain function and, therefore, quality of life in older populations who tend to sleep longer as they age.

### Electronic supplementary material

Below is the link to the electronic supplementary material.

Supplementary Material 1

Supplementary Material 2

Supplementary Material 3

Supplementary Material 4

## Data Availability

The datasets analyzed during the current study are not publicly available due to a lack of consent from the participants to share the data, but are available from the corresponding author upon reasonable request.

## List of abbreviations

BMI: body mass index
CES-D: Center for Epidemiologic Studies Depression Scale
CIs: confidence intervals
GEE: generalized estimating equation
MET: metabolic equivalent
MMSE: Mini-Mental State Examination
NILS-LSA: National Institute for Longevity Sciences-Longitudinal Study of Aging
ORs: odds ratios
Q: quartiles
SD: standard deviation
