# Supplementary material for: Dietary amino acid intake and sleep duration are additively involved in future cognitive decline in Japanese adults aged 60 years or over: a community-based longitudinal study
Source: BMC Geriatr. 2023 Oct 11;23:653. doi: 10.1186/s12877-023-04359-2 (PMC10568860; doi:10.1186/s12877-023-04359-2)
Supplement: Supplementary file 1 — Supplementary Material 1 [file 12877_2023_4359_MOESM1_ESM.docx]

**Additional File 1**

**Supplemental Table 1**

**File format:** Microsoft word (.docx)

**Title of data:** Multivariable-adjusted longitudinal association of the combined groups of amino acid intake and sleep duration with incidence of cognitive impairment

**Description of data:** Supplemental Table 1 shows the results of an additional analysis. This analysis was performed to verify the association between amino acid intake and incidence of cognitive impairment in short to moderate sleepers (≤8 h) and long-sleepers (>8 h). Participants were classified into four groups based on amino acid intake (i.e., low- and middle to high-intake) and sleep duration (i.e., ≤8 h and >8 h): low-intake with >8 h sleep, low-intake with ≤8 h sleep, middle to high-intake with >8 h sleep, and middle to high-intake with ≤8 h sleep. Using the middle to high-intake with ≤8 h sleep group as the reference, the ORs and 95% CIs for cognitive impairment were estimated using the generalized estimating equation.

**Supplemental Table 1.**

**Multivariable-adjusted longitudinal association of the combined groups of amino acid intake and sleep duration with incidence of cognitive impairment**

|  | Crude* | | |  | Model 1* | | |  | Model 2* | | |  | Model 3* | | |
| --- | --- | --- | --- | --- | --- | --- | --- | --- | --- | --- | --- | --- | --- | --- | --- |
| Amino acids and sleep group^†^ | OR | 95 %CI | P value |  | OR | 95 %CI | P value |  | OR | 95 %CI | P value |  | OR | 95 %CI | P value |
| Ile (low) x Sleep (>8 h) | 2.30 | 1.57–3.38 | <0.001 |  | 1.81 | 1.20–2.73 | 0.005 |  | 1.92 | 1.25–2.96 | 0.003 |  | 2.11 | 1.30–3.43 | 0.002 |
| Ile (low) x Sleep (≤8 h) | 1.51 | 1.10–2.06 | 0.010 |  | 1.43 | 1.02–2.00 | 0.038 |  | 1.42 | 1.01–1.99 | 0.042 |  | 1.45 | 1.03–2.02 | 0.031 |
| Ile (middle to high) x Sleep (>8 h) | 1.35 | 0.94–1.95 | 0.107 |  | 1.32 | 0.90–1.95 | 0.160 |  | 1.42 | 0.93–2.16 | 0.107 |  | 1.55 | 0.99–2.42 | 0.055 |
| Ile (middle to high) x Sleep (≤8 h) | Ref. |  |  |  | Ref. |  |  |  | Ref. |  |  |  | Ref. |  |  |
| Leu (low) x Sleep (>8 h) | 2.32 | 1.59–3.39 | <0.001 |  | 1.84 | 1.23–2.77 | 0.003 |  | 1.98 | 1.29–3.03 | 0.002 |  | 2.22 | 1.37–3.58 | 0.001 |
| Leu (low) x Sleep (≤8 h) | 1.50 | 1.10–2.06 | 0.011 |  | 1.43 | 1.02–2.01 | 0.038 |  | 1.42 | 1.01–2.00 | 0.043 |  | 1.45 | 1.04–2.04 | 0.030 |
| Leu (middle to high) x Sleep (>8 h) | 1.37 | 0.95–1.98 | 0.094 |  | 1.37 | 0.93–2.02 | 0.108 |  | 1.49 | 0.98–2.28 | 0.064 |  | 1.67 | 1.06–2.61 | 0.026 |
| Leu (middle to high) x Sleep (≤8 h) | Ref. |  |  |  | Ref. |  |  |  | Ref. |  |  |  | Ref. |  |  |
| Lys (low) x Sleep (>8 h) | 2.14 | 1.45–3.16 | <0.001 |  | 1.68 | 1.12–2.54 | 0.013 |  | 1.74 | 1.14–2.65 | 0.010 |  | 1.88 | 1.17–3.04 | 0.010 |
| Lys (low) x Sleep (≤8 h) | 1.54 | 1.13–2.10 | 0.007 |  | 1.47 | 1.05–2.06 | 0.024 |  | 1.47 | 1.05–2.05 | 0.026 |  | 1.48 | 1.06–2.07 | 0.021 |
| Lys (middle to high) x Sleep (>8 h) | 1.31 | 0.91–1.89 | 0.146 |  | 1.31 | 0.89–1.94 | 0.171 |  | 1.37 | 0.90–2.07 | 0.139 |  | 1.48 | 0.94–2.32 | 0.087 |
| Lys (middle to high) x Sleep (≤8 h) | Ref. |  |  |  | Ref. |  |  |  | Ref. |  |  |  | Ref. |  |  |
| Met (low) x Sleep (>8 h) | 2.07 | 1.41–3.03 | <0.001 |  | 1.64 | 1.09–2.47 | 0.017 |  | 1.67 | 1.09–2.55 | 0.018 |  | 1.71 | 1.06–2.75 | 0.027 |
| Met (low) x Sleep (≤8 h) | 1.47 | 1.08–2.01 | 0.015 |  | 1.41 | 1.01–1.98 | 0.046 |  | 1.41 | 1.00–1.97 | 0.049 |  | 1.41 | 1.01–1.98 | 0.044 |
| Met (middle to high) x Sleep (>8 h) | 1.09 | 0.75–1.59 | 0.653 |  | 1.13 | 0.76–1.69 | 0.549 |  | 1.15 | 0.76–1.76 | 0.508 |  | 1.18 | 0.75–1.87 | 0.479 |
| Met (middle to high) x Sleep (≤8 h) | Ref. |  |  |  | Ref. |  |  |  | Ref. |  |  |  | Ref. |  |  |
| Cys (low) x Sleep (>8 h) | 2.48 | 1.66–3.70 | <0.001 |  | 1.91 | 1.24–2.93 | 0.003 |  | 2.03 | 1.28–3.21 | 0.003 |  | 2.15 | 1.29–3.57 | 0.003 |
| Cys (low) x Sleep (≤8 h) | 1.40 | 1.03–1.90 | 0.032 |  | 1.33 | 0.96–1.85 | 0.091 |  | 1.32 | 0.95–1.84 | 0.100 |  | 1.34 | 0.96–1.87 | 0.081 |
| Cys (middle to high) x Sleep (>8 h) | 1.23 | 0.85–1.79 | 0.267 |  | 1.18 | 0.80–1.74 | 0.397 |  | 1.26 | 0.82–1.93 | 0.284 |  | 1.32 | 0.85–2.07 | 0.217 |
| Cys (middle to high) x Sleep (≤8 h) | Ref. |  |  |  | Ref. |  |  |  | Ref. |  |  |  | Ref. |  |  |
| Phe (low) x Sleep (>8 h) | 2.43 | 1.64–3.60 | <0.001 |  | 1.89 | 1.25–2.87 | 0.003 |  | 2.06 | 1.33–3.21 | 0.001 |  | 2.34 | 1.43–3.83 | 0.001 |
| Phe (low) x Sleep (≤8 h) | 1.51 | 1.11–2.05 | 0.009 |  | 1.43 | 1.02–2.00 | 0.037 |  | 1.42 | 1.01–1.98 | 0.041 |  | 1.46 | 1.04–2.03 | 0.027 |
| Phe (middle to high) x Sleep (>8 h) | 1.41 | 0.98–2.04 | 0.067 |  | 1.37 | 0.93–2.02 | 0.110 |  | 1.51 | 0.99–2.30 | 0.057 |  | 1.69 | 1.08–2.64 | 0.022 |
| Phe (middle to high) x Sleep (≤8 h) | Ref. |  |  |  | Ref. |  |  |  | Ref. |  |  |  | Ref. |  |  |
| Tyr (low) x Sleep (>8 h) | 2.29 | 1.57–3.36 | <0.001 |  | 1.77 | 1.18–2.68 | 0.006 |  | 1.88 | 1.22–2.90 | 0.004 |  | 2.06 | 1.27–3.36 | 0.004 |
| Tyr (low) x Sleep (≤8 h) | 1.50 | 1.10–2.05 | 0.011 |  | 1.43 | 1.03–2.00 | 0.035 |  | 1.43 | 1.02–2.00 | 0.038 |  | 1.45 | 1.04–2.03 | 0.029 |
| Tyr (middle to high) x Sleep (>8 h) | 1.33 | 0.92–1.92 | 0.135 |  | 1.30 | 0.88–1.91 | 0.185 |  | 1.39 | 0.91–2.13 | 0.127 |  | 1.52 | 0.97–2.39 | 0.069 |
| Tyr (middle to high) x Sleep (≤8 h) | Ref. |  |  |  | Ref. |  |  |  | Ref. |  |  |  | Ref. |  |  |
| Thr (low) x Sleep (>8 h) | 2.18 | 1.48–3.22 | <0.001 |  | 1.71 | 1.13–2.58 | 0.012 |  | 1.78 | 1.16–2.75 | 0.009 |  | 1.93 | 1.18–3.15 | 0.008 |
| Thr (low) x Sleep (≤8 h) | 1.53 | 1.13–2.09 | 0.007 |  | 1.45 | 1.04–2.03 | 0.028 |  | 1.45 | 1.04–2.03 | 0.030 |  | 1.47 | 1.05–2.05 | 0.024 |
| Thr (middle to high) x Sleep (>8 h) | 1.30 | 0.90–1.88 | 0.168 |  | 1.28 | 0.87–1.89 | 0.214 |  | 1.35 | 0.89–2.05 | 0.164 |  | 1.45 | 0.92–2.29 | 0.105 |
| Thr (middle to high) x Sleep (≤ h) | Ref. |  |  |  | Ref. |  |  |  | Ref. |  |  |  | Ref. |  |  |
| Trp (low) x Sleep (>8 h) | 2.35 | 1.58–3.49 | <0.001 |  | 1.79 | 1.18–2.73 | 0.007 |  | 1.91 | 1.23–2.97 | 0.004 |  | 2.12 | 1.30–3.47 | 0.003 |
| Trp (low) x Sleep (≤8 h) | 1.53 | 1.12–2.08 | 0.007 |  | 1.47 | 1.05–2.05 | 0.024 |  | 1.46 | 1.04–2.04 | 0.027 |  | 1.49 | 1.07–2.08 | 0.018 |
| Trp (middle to high) x Sleep (>8 h) | 1.40 | 0.97–2.01 | 0.070 |  | 1.36 | 0.93–2.00 | 0.116 |  | 1.47 | 0.97–2.22 | 0.071 |  | 1.61 | 1.04–2.51 | 0.034 |
| Trp (middle to high) x Sleep (≤8 h) | Ref. |  |  |  | Ref. |  |  |  | Ref. |  |  |  | Ref. |  |  |
| Val (low) x Sleep (>8 h) | 2.33 | 1.60–3.41 | <0.001 |  | 1.84 | 1.22–2.76 | 0.004 |  | 1.96 | 1.27–3.01 | 0.002 |  | 2.16 | 1.34–3.50 | 0.002 |
| Val (low) x Sleep (≤8 h) | 1.48 | 1.08–2.03 | 0.014 |  | 1.41 | 1.01–1.98 | 0.047 |  | 1.40 | 1.00–1.97 | 0.052 |  | 1.43 | 1.02–2.00 | 0.038 |
| Val (middle to high) x Sleep (>8 h) | 1.32 | 0.91–1.92 | 0.1450 |  | 1.31 | 0.88–1.94 | 0.178 |  | 1.41 | 0.92–2.17 | 0.115 |  | 1.55 | 0.98–2.44 | 0.058 |
| Val (middle to high) x Sleep (≤8 h) | Ref. |  |  |  | Ref. |  |  |  | Ref. |  |  |  | Ref. |  |  |
| His (low) x Sleep (>8 h) | 1.96 | 1.31–2.94 | 0.001 |  | 1.51 | 0.98–2.33 | 0.061 |  | 1.52 | 0.98–2.37 | 0.062 |  | 1.53 | 0.93–2.51 | 0.091 |
| His (low) x Sleep (≤8 h) | 1.55 | 1.15–2.10 | 0.005 |  | 1.49 | 1.07–2.07 | 0.019 |  | 1.49 | 1.07–2.07 | 0.019 |  | 1.49 | 1.07–2.07 | 0.018 |
| His (middle to high) x Sleep (>8 h) | 1.14 | 0.79–1.65 | 0.495 |  | 1.15 | 0.78–1.70 | 0.477 |  | 1.16 | 0.77–1.76 | 0.473 |  | 1.17 | 0.75–1.82 | 0.492 |
| His (middle to high) x Sleep (≤8 h) | Ref. |  |  |  | Ref. |  |  |  | Ref. |  |  |  | Ref. |  |  |
| Arg (low) x Sleep (>8 h) | 2.31 | 1.55–3.45 | <0.001 |  | 1.82 | 1.20–2.75 | 0.005 |  | 1.90 | 1.24–2.92 | 0.003 |  | 2.04 | 1.26–3.29 | 0.004 |
| Arg (low) x Sleep (≤8 h) | 1.48 | 1.09–2.00 | 0.013 |  | 1.39 | 0.99–1.94 | 0.054 |  | 1.38 | 0.99–1.94 | 0.057 |  | 1.41 | 1.01–1.96 | 0.045 |
| Arg (middle to high) x Sleep (>8 h) | 1.23 | 0.84–1.78 | 0.289 |  | 1.22 | 0.83–1.79 | 0.323 |  | 1.28 | 0.85–1.93 | 0.245 |  | 1.36 | 0.88–2.11 | 0.166 |
| Arg (middle to high) x Sleep (≤8 h) | Ref. |  |  |  | Ref. |  |  |  | Ref. |  |  |  | Ref. |  |  |
| Ala (low) x Sleep (>8 h) | 2.27 | 1.55–3.33 | <0.001 |  | 1.76 | 1.18–2.63 | 0.006 |  | 1.86 | 1.22–2.82 | 0.004 |  | 2.06 | 1.29–3.29 | 0.003 |
| Ala (low) x Sleep (≤8 h) | 1.53 | 1.12–2.09 | 0.007 |  | 1.47 | 1.05–2.06 | 0.024 |  | 1.46 | 1.04–2.05 | 0.028 |  | 1.49 | 1.07–2.09 | 0.019 |
| Ala (middle to high) x Sleep (>8 h) | 1.40 | 0.97–2.03 | 0.075 |  | 1.39 | 0.94–2.05 | 0.096 |  | 1.48 | 0.98–2.23 | 0.064 |  | 1.64 | 1.05–2.55 | 0.029 |
| Ala (middle to high) x Sleep (≤8 h) | Ref. |  |  |  | Ref. |  |  |  | Ref. |  |  |  | Ref. |  |  |
| Asp (low) x Sleep (>8 h) | 2.39 | 1.61–3.54 | <0.001 |  | 1.78 | 1.18–2.67 | 0.006 |  | 1.84 | 1.21–2.82 | 0.005 |  | 1.96 | 1.22–3.17 | 0.006 |
| Asp (low) x Sleep (≤8 h) | 1.45 | 1.06–1.97 | 0.019 |  | 1.38 | 0.98–1.94 | 0.061 |  | 1.38 | 0.98–1.93 | 0.065 |  | 1.39 | 0.99–1.96 | 0.055 |
| Asp (middle to high) x Sleep (>8 h) | 1.26 | 0.87–1.81 | 0.222 |  | 1.20 | 0.81–1.76 | 0.363 |  | 1.25 | 0.82–1.89 | 0.295 |  | 1.33 | 0.85–2.07 | 0.212 |
| Asp (middle to high) x Sleep (≤8 h) | Ref. |  |  |  | Ref. |  |  |  | Ref. |  |  |  | Ref. |  |  |
| Glu (low) x Sleep (>8 h) | 2.00 | 1.33–3.01 | 0.001 |  | 1.53 | 1.01–2.32 | 0.047 |  | 1.53 | 0.99–2.38 | 0.058 |  | 1.50 | 0.93–2.43 | 0.095 |
| Glu (low) x Sleep (≤8 h) | 1.54 | 1.13–2.08 | 0.006 |  | 1.43 | 1.03–1.99 | 0.032 |  | 1.43 | 1.03–1.99 | 0.033 |  | 1.43 | 1.03–1.98 | 0.033 |
| Glu (middle to high) x Sleep (>8 h) | 1.14 | 0.79–1.66 | 0.486 |  | 1.05 | 0.71–1.57 | 0.793 |  | 1.06 | 0.69–1.63 | 0.796 |  | 1.04 | 0.67–1.61 | 0.861 |
| Glu (middle to high) x Sleep (≤8 h) | Ref. |  |  |  | Ref. |  |  |  | Ref. |  |  |  | Ref. |  |  |
| Gly (low) x Sleep (>8 h) | 2.21 | 1.47–3.31 | <0.001 |  | 1.70 | 1.12–2.60 | 0.013 |  | 1.79 | 1.15–2.77 | 0.010 |  | 1.92 | 1.18–3.13 | 0.009 |
| Gly (low) x Sleep (≤8 h) | 1.53 | 1.13–2.07 | 0.006 |  | 1.46 | 1.05–2.03 | 0.023 |  | 1.45 | 1.05–2.02 | 0.026 |  | 1.47 | 1.06–2.04 | 0.020 |
| Gly (middle to high) x Sleep (>8 h) | 1.31 | 0.90–1.90 | 0.157 |  | 1.31 | 0.89–1.92 | 0.166 |  | 1.38 | 0.91–20.9 | 0.127 |  | 1.48 | 0.96–2.28 | 0.078 |
| Gly (middle to high) x Sleep (≤8 h) | Ref. |  |  |  | Ref. |  |  |  | Ref. |  |  |  | Ref. |  |  |
| Pro (low) x Sleep (>8 h) | 2.37 | 1.60–3.51 | <0.001 |  | 1.87 | 1.24–2.83 | 0.003 |  | 1.93 | 1.26–2.97 | 0.003 |  | 1.97 | 1.25–3.13 | 0.004 |
| Pro (low) x Sleep (≤8 h) | 1.45 | 1.07–1.98 | 0.018 |  | 1.32 | 0.94–1.84 | 0.105 |  | 1.32 | 0.94–1.84 | 0.106 |  | 1.33 | 0.95–1.85 | 0.097 |
| Pro (middle to high) x Sleep (>8 h) | 1.25 | 0.87–1.80 | 0.229 |  | 1.11 | 0.75–1.63 | 0.608 |  | 1.15 | 0.75–1.75 | 0.517 |  | 1.16 | 0.77–1.77 | 0.476 |
| Pro (middle to high) x Sleep (≤8 h) | Ref. |  |  |  | Ref. |  |  |  | Ref. |  |  |  | Ref. |  |  |
| Ser (low) x Sleep (>8 h) | 2.58 | 1.75–3.81 | <0.001 |  | 1.96 | 1.30–2.97 | 0.001 |  | 2.11 | 1.36–3.28 | 0.001 |  | 2.33 | 1.42–3.81 | 0.001 |
| Ser (low) x Sleep (≤8 h) | 1.42 | 1.04–1.93 | 0.027 |  | 1.36 | 0.97–1.90 | 0.074 |  | 1.35 | 0.96–1.89 | 0.081 |  | 1.38 | 0.99–1.93 | 0.059 |
| Ser (middle to high) x Sleep (>8 h) | 1.28 | 0.88–1.84 | 0.193 |  | 1.25 | 0.85–1.84 | 0.249 |  | 1.36 | 0.89–2.07 | 0.154 |  | 1.48 | 0.95–2.32 | 0.087 |
| Ser (middle to high) x Sleep (≤8 h) | Ref. |  |  |  | Ref. |  |  |  | Ref. |  |  |  | Ref. |  |  |
| Hyp (low) x Sleep (>8 h) | 1.83 | 1.24–2.71 | 0.003 |  | 1.52 | 1.00–2.29 | 0.048 |  | 1.52 | 1.00–2.31 | 0.049 |  | 1.54 | 0.98–2.41 | 0.062 |
| Hyp (low) x Sleep (≤8 h) | 1.65 | 1.22–2.25 | 0.001 |  | 1.56 | 1.11–2.20 | 0.010 |  | 1.56 | 1.12–2.20 | 0.010 |  | 1.57 | 1.12–2.20 | 0.009 |
| Hyp (middle to high) x Sleep (>8 h) | 1.22 | 0.85–1.75 | 0.292 |  | 1.31 | 0.89–1.93 | 0.166 |  | 1.32 | 0.89–1.95 | 0.163 |  | 1.33 | 0.89–1.99 | 0.169 |
| Hyp (middle to high) x Sleep (≤8 h) | Ref. |  |  |  | Ref. |  |  |  | Ref. |  |  |  | Ref. |  |  |

*ORs and 95% CIs estimated using the generalized estimating equations. ^†^Amino acid groups: based on the sex-stratified quartiles of amino acid intake, quartile 1 was defined as the low intake group and quartiles 2 to 4 were defined as the middle to high intake group.

Model 1: adjusted for sex, age (60-69 y/70-79 y/≥80 y), BMI (kg/m²), MMSE (score), CES-D (score), education (0-7 y/8-15 y/≥16 y), smoking status (current/not), employment status (yes/no), using of hypnotics, sedatives, or anxiolytics (yes/no), physical activity (MET-min/d), history of stroke, hypertension, ischemic heart disease, dyslipidemia, and diabetes mellitus at baseline, and follow-up period (y).

Model 2: adjusted for energy intake (kcal/d) in addition to the variables in model 1.

Model 3: adjusted for protein intake (g/d) in addition to the variables in model 1.

OR, odds ratio; CI, confidence interval; Ile, Isoleucine; Leu, Leucine; Lys, Lysine; Met, Methionine; Cys, Cystine; Phe, Phenylalanine; Tyr, Tyrosine; Thr, Threonine; Trp, Tryptophan; Val, Valine; His, Histidine; Arg, Arginine; Ala, Alanine; Asp, Aspartic acid; Glu, Glutamic acid; Gly, Glycine; Pro, Proline; Ser, Serine; Hyp, Hydroxyproline; BMI, body mass index; MMSE, Mini-Mental State Examination; CES-D, The Center for Epidemiologic Studies Depression Scale; MET, metabolic equivalents.
