# Supplementary material for: Dietary amino acid intake and sleep duration are additively involved in future cognitive decline in Japanese adults aged 60 years or over: a community-based longitudinal study
Source: BMC Geriatr. 2023 Oct 11;23:653. doi: 10.1186/s12877-023-04359-2 (PMC10568860; doi:10.1186/s12877-023-04359-2)
Supplement: Supplementary file 2 — Supplementary Material 2 [file 12877_2023_4359_MOESM2_ESM.docx]

**Additional File 2**

**Supplemental Table 2**

**File format:** Microsoft word (.docx)

**Title of data:** Food consumption (g/100 kcal/d) according to the low- and middle to high-intake groups of cystine, proline, and serine in long sleepers

**Description of data:** Supplemental Table 2 shows the results of a supplemental analysis. In this analysis, comparisons of food consumption (g/100 kcal/day) between the low- and the middle to high-intake groups of cystine, proline, and serine were analyzed using the t-test. Cystine, proline, and serine were selected based on the significant association between amino acid intake and the incidence of cognitive impairment calculated using the generalized estimating equation.

**Supplemental Table 2.**

**Food consumption (g/100 kcal/d) according to the low- and middle to high-intake groups of cystine, proline, and serine in long sleepers^*^**

|  | Cystine^†^ | | | |  | Proline^†^ | | | |  | Serine^†^ | | | |
| --- | --- | --- | --- | --- | --- | --- | --- | --- | --- | --- | --- | --- | --- | --- |
|  | Low |  | Middle to high |  |  | Low |  | Middle to high |  |  | Low |  | Middle to high |  |
| Range (mg/day): Male | 755.0–1038.6 |  | 1049.3–1746.0 |  |  | 2491.9–3739.4 |  | 3754.9–6020.4 |  |  | 2209.8–3207.4 |  | 3227.7–5793.6 |  |
| Female | 567.0–864.0 |  | 872.3–1445.0 |  |  | 1738.5–3295.1 |  | 3314.8–5894.5 |  |  | 1677.5–2685.9 |  | 2696.1–5059.1 |  |
|  | (n= 59) |  | (n= 118) | P value |  | (n= 59) |  | (n= 118) | P value |  | (n= 56) |  | (n= 121) | P value |
| Cereal grains | 25.2 ± 6.4 |  | 21.7 ± 4.8 | <0.001 |  | 25.6 ± 6.4 |  | 21.5 ± 4.6 | <0.001 |  | 25.6 ± 6.2 |  | 21.5 ± 4.8 | <0.001 |
| Tubers and roots | 2.6 ± 2.0 |  | 2.2 ± 1.7 | 0.176 |  | 2.5 ± 1.7 |  | 2.3 ± 1.8 | 0.344 |  | 2.5 ± 1.9 |  | 2.3 ± 1.8 | 0.412 |
| Sugars and sweeteners | 0.6 ± 0.6 |  | 0.5 ± 0.4 | 0.066 |  | 0.6 ± 0.6 |  | 0.5 ± 0.5 | 0.102 |  | 0.6 ± 0.6 |  | 0.5 ± 0.4 | 0.090 |
| Beans and legumes | 2.7 ± 1.7 |  | 3.7 ± 2.5 | 0.006 |  | 3.1 ± 2.7 |  | 3.5 ± 2.1 | 0.326 |  | 2.4 ± 1.7 |  | 3.8 ± 2.5 | <0.001 |
| Nuts and seeds | 0.2 ± 0.3 |  | 0.2 ± 0.4 | 0.415 |  | 0.1 ± 0.3 |  | 0.2 ± 0.4 | 0.088 |  | 0.1 ± 0.3 |  | 0.2 ± 0.4 | 0.126 |
| Vegetables | 8.5 ± 4.2 |  | 9.9 ± 4.7 | 0.060 |  | 9.0 ± 4.2 |  | 9.6 ± 4.8 | 0.406 |  | 8.0 ± 4.2 |  | 10.0 ± 4.6 | 0.007 |
| β-carotene-rich vegetables | 6.4 ± 4.0 |  | 6.9 ± 3.9 | 0.409 |  | 6.4 ± 4.2 |  | 6.9 ± 3.8 | 0.412 |  | 6.3 ± 4.2 |  | 6.9 ± 3.8 | 0.309 |
| Fruits | 8.2 ± 6.3 |  | 8.1 ± 5.7 | 0.859 |  | 7.3 ± 6.3 |  | 8.6 ± 5.6 | 0.170 |  | 8.6 ± 7.1 |  | 7.9 ± 5.2 | 0.427 |
| Mushrooms | 0.5 ± 0.5 |  | 0.6 ± 0.6 | 0.123 |  | 0.5 ± 0.5 |  | 0.6 ± 0.6 | 0.448 |  | 0.4 ± 0.5 |  | 0.6 ± 0.6 | 0.062 |
| Seaweed | 0.9 ± 0.9 |  | 0.8 ± 0.9 | 0.563 |  | 0.9 ± 1.0 |  | 0.8 ± 0.9 | 0.536 |  | 0.8 ± 0.9 |  | 0.9 ± 0.9 | 0.757 |
| Fish and seafood | 4.5 ± 2.3 |  | 5.1 ± 2.5 | 0.105 |  | 4.7 ± 2.3 |  | 5.0 ± 2.5 | 0.387 |  | 4.2 ± 2.3 |  | 5.2 ± 2.4 | 0.010 |
| Meat | 2.1 ± 1.6 |  | 2.7 ± 1.7 | 0.029 |  | 2.3 ± 1.6 |  | 2.6 ± 1.7 | 0.243 |  | 2.4 ± 2.0 |  | 2.6 ± 1.5 | 0.457 |
| Eggs | 1.7 ± 0.9 |  | 2.2 ± 1.1 | 0.004 |  | 2.1 ± 1.1 |  | 2.0 ± 1.0 | 0.446 |  | 1.9 ± 1.1 |  | 2.1 ± 1.0 | 0.174 |
| Milk and dairy products | 8.3 ± 7.2 |  | 7.2 ± 5.5 | 0.282 |  | 4.6 ± 5.2 |  | 9.0 ± 6.0 | <.0001 |  | 6.8 ± 6.7 |  | 7.9 ± 5.8 | 0.237 |
| Fats and oils | 0.4 ± 0.2 |  | 0.4 ± 0.3 | 0.372 |  | 0.4 ± 0.3 |  | 0.4 ± 0.3 | 0.550 |  | 0.4 ± 0.3 |  | 0.4 ± 0.3 | 0.721 |
| Confectioneries | 1.4 ± 1.8 |  | 1.8 ± 1.7 | 0.174 |  | 1.5 ± 1.9 |  | 1.8 ± 1.6 | 0.260 |  | 1.4 ± 1.8 |  | 1.8 ± 1.7 | 0.153 |
| Beverages | 37.5 ± 19.6 |  | 39.7 ± 18.9 | 0.469 |  | 39.9 ± 19.3 |  | 38.6 ± 19.1 | 0.661 |  | 36.0 ± 19.1 |  | 40.4 ± 19.0 | 0.161 |
| Seasoning and spices | 3.5 ± 1.4 |  | 3.2 ± 1.0 | 0.075 |  | 3.4 ± 1.1 |  | 3.3 ± 1.2 | 0.363 |  | 3.5 ± 1.4 |  | 3.2 ± 1.0 | 0.251 |

Data are presented as mean ± standard deviation. ^*^Categorized by sleep duration >8 h per day. ^†^Based on the sex-stratified quartiles of amino acid intake, quartile 1 was defined as the low intake group and quartiles 2 to 4 were defined as the middle to high intake group.
