# Supplementary material for: Dietary amino acid intake and sleep duration are additively involved in future cognitive decline in Japanese adults aged 60 years or over: a community-based longitudinal study
Source: BMC Geriatr. 2023 Oct 11;23:653. doi: 10.1186/s12877-023-04359-2 (PMC10568860; doi:10.1186/s12877-023-04359-2)
Supplement: Supplementary file 3 — Supplementary Material 3 [file 12877_2023_4359_MOESM3_ESM.docx]

**Additional File 3**

**Supplemental Table 3**

**File format:** Microsoft word (.docx)

**Title of data:** Multivariable-adjusted longitudinal association between amino acid intake and incidence of cognitive impairment in long-sleepers

**Description of data:** Supplemental Table 3 shows the results of a supplemental analysis. This analysis was performed to examine the dose-response relationship between amino acid intake and cognitive function; the ORs and 95% CIs for incidence of cognitive impairment in the Q2 to Q4 amino acid intake groups were estimated in long sleepers, using the Q1 amino acid intake group as a reference.

**Supplemental Table 3.**

**Multivariable-adjusted longitudinal association between amino acid intakes and incidence of cognitive impairment in long-sleepers**

|  | Crude* | | |  | Model 1* | | |  | Model 2* | | |  | Model 3* | | |
| --- | --- | --- | --- | --- | --- | --- | --- | --- | --- | --- | --- | --- | --- | --- | --- |
|  | OR | 95 %CI | P value |  | OR | 95 %CI | P value |  | OR | 95 %CI | P value |  | OR | 95 %CI | P value |
| Ile Q1 | Ref. |  |  |  | Ref. |  |  |  | Ref. |  |  |  | Ref. |  |  |
| Ile Q2 | 0.73 | 0.42–1.26 | 0.256 |  | 0.69 | 0.40–1.21 | 0.199 |  | 0.70 | 0.38–1.29 | 0.254 |  | 0.61 | 0.32–1.16 | 0.132 |
| Ile Q3 | 0.55 | 0.30–1.00 | 0.049 |  | 0.68 | 0.34–1.34 | 0.264 |  | 0.68 | 0.33–1.43 | 0.310 |  | 0.54 | 0.21–1.40 | 0.203 |
| Ile Q4 | 0.69 | 0.40–1.19 | 0.178 |  | 0.93 | 0.52–1.65 | 0.796 |  | 0.94 | 0.46–1.94 | 0.869 |  | 0.66 | 0.23–1.92 | 0.445 |
| Leu Q1 | Ref. |  |  |  | Ref. |  |  |  | Ref. |  |  |  | Ref. |  |  |
| Leu Q2 | 0.71 | 0.41–1.24 | 0.235 |  | 0.68 | 0.38–1.21 | 0.188 |  | 0.68 | 0.36–1.28 | 0.235 |  | 0.60 | 0.31–1.15 | 0.123 |
| Leu Q3 | 0.55 | 0.31–0.99 | 0.047 |  | 0.66 | 0.34–1.29 | 0.222 |  | 0.66 | 0.32–1.36 | 0.264 |  | 0.53 | 0.21–1.35 | 0.183 |
| Leu Q4 | 0.68 | 0.40–1.18 | 0.170 |  | 0.94 | 0.53–1.65 | 0.822 |  | 0.95 | 0.47–1.92 | 0.880 |  | 0.68 | 0.24–1.91 | 0.460 |
| Lys Q1 | Ref. |  |  |  | Ref. |  |  |  | Ref. |  |  |  | Ref. |  |  |
| Lys Q2 | 0.74 | 0.42–1.30 | 0.291 |  | 0.75 | 0.43–1.32 | 0.319 |  | 0.78 | 0.43–1.39 | 0.394 |  | 0.74 | 0.39–1.43 | 0.376 |
| Lys Q3 | 0.72 | 0.41–1.24 | 0.235 |  | 0.91 | 0.45–1.84 | 0.793 |  | 0.96 | 0.46–1.99 | 0.907 |  | 0.89 | 0.34–2.33 | 0.816 |
| Lys Q4 | 0.73 | 0.41–1.28 | 0.267 |  | 0.99 | 0.57–1.72 | 0.961 |  | 1.06 | 0.54–2.05 | 0.874 |  | 0.96 | 0.34–2.72 | 0.937 |
| Met Q1 | Ref. |  |  |  | Ref. |  |  |  | Ref. |  |  |  | Ref. |  |  |
| Met Q2 | 0.72 | 0.41–1.24 | 0.230 |  | 0.73 | 0.42–1.27 | 0.270 |  | 0.78 | 0.42–1.42 | 0.412 |  | 0.80 | 0.42–1.53 | 0.502 |
| Met Q3 | 0.58 | 0.32–1.06 | 0.076 |  | 0.63 | 0.29–1.38 | 0.247 |  | 0.70 | 0.30–1.60 | 0.395 |  | 0.74 | 0.27–2.06 | 0.565 |
| Met Q4 | 0.86 | 0.50–1.49 | 0.593 |  | 1.29 | 0.71–2.34 | 0.394 |  | 1.48 | 0.71–3.06 | 0.293 |  | 1.64 | 0.56–4.85 | 0.369 |
| Cys Q1 | Ref. |  |  |  | Ref. |  |  |  | Ref. |  |  |  | Ref. |  |  |
| Cys Q2 | 0.55 | 0.32–0.96 | 0.034 |  | 0.61 | 0.35–1.08 | 0.092 |  | 0.59 | 0.32–1.08 | 0.089 |  | 0.47 | 0.25–0.89 | 0.020 |
| Cys Q3 | 0.58 | 0.32–1.04 | 0.068 |  | 0.64 | 0.31–1.30 | 0.214 |  | 0.59 | 0.28–1.27 | 0.177 |  | 0.42 | 0.17–1.01 | 0.054 |
| Cys Q4 | 0.58 | 0.33–1.01 | 0.055 |  | 0.79 | 0.46–1.36 | 0.393 |  | 0.71 | 0.35–1.44 | 0.346 |  | 0.41 | 0.16–1.06 | 0.066 |
| Phe Q1 | Ref. |  |  |  | Ref. |  |  |  | Ref. |  |  |  | Ref. |  |  |
| Phe Q2 | 0.68 | 0.39–1.18 | 0.167 |  | 0.68 | 0.38–1.22 | 0.200 |  | 0.67 | 0.36–1.26 | 0.215 |  | 0.55 | 0.28–1.07 | 0.079 |
| Phe Q3 | 0.55 | 0.30–1.00 | 0.052 |  | 0.65 | 0.33–1.29 | 0.217 |  | 0.63 | 0.30–1.35 | 0.238 |  | 0.46 | 0.19–1.13 | 0.090 |
| Phe Q4 | 0.63 | 0.37–1.08 | 0.095 |  | 0.87 | 0.49–1.54 | 0.633 |  | 0.84 | 0.41–1.75 | 0.646 |  | 0.51 | 0.19–1.41 | 0.196 |
| Tyr Q1 | Ref. |  |  |  | Ref. |  |  |  | Ref. |  |  |  | Ref. |  |  |
| Tyr Q2 | 0.77 | 0.45–1.32 | 0.346 |  | 0.79 | 0.45–1.36 | 0.387 |  | 0.80 | 0.44–1.45 | 0.458 |  | 0.72 | 0.37–1.41 | 0.341 |
| Tyr Q3 | 0.39 | 0.21–0.73 | 0.003 |  | 0.43 | 0.21–0.87 | 0.019 |  | 0.44 | 0.21–0.95 | 0.037 |  | 0.38 | 0.14–1.00 | 0.050 |
| Tyr Q4 | 0.81 | 0.47–1.40 | 0.452 |  | 1.15 | 0.63–2.09 | 0.649 |  | 1.19 | 0.57–2.48 | 0.639 |  | 0.94 | 0.28–3.15 | 0.924 |
| Thr Q1 | Ref. |  |  |  | Ref. |  |  |  | Ref. |  |  |  | Ref. |  |  |
| Thr Q2 | 0.73 | 0.42–1.26 | 0.256 |  | 0.75 | 0.43–1.28 | 0.289 |  | 0.77 | 0.43–1.38 | 0.380 |  | 0.71 | 0.37–1.37 | 0.308 |
| Thr Q3 | 0.67 | 0.37–1.22 | 0.190 |  | 0.77 | 0.37–1.59 | 0.476 |  | 0.81 | 0.38–1.75 | 0.593 |  | 0.71 | 0.26–1.96 | 0.510 |
| Thr Q4 | 0.73 | 0.42–1.27 | 0.264 |  | 0.99 | 0.56–1.78 | 0.984 |  | 1.07 | 0.52–2.17 | 0.860 |  | 0.88 | 0.29–2.71 | 0.829 |
| Trp Q1 | Ref. |  |  |  | Ref. |  |  |  | Ref. |  |  |  | Ref. |  |  |
| Trp Q2 | 0.77 | 0.45–1.33 | 0.352 |  | 0.81 | 0.46–1.43 | 0.470 |  | 0.83 | 0.45–1.51 | 0.535 |  | 0.71 | 0.37–1.36 | 0.306 |
| Trp Q3 | 0.52 | 0.29–0.94 | 0.029 |  | 0.61 | 0.31–1.16 | 0.132 |  | 0.62 | 0.31–1.24 | 0.175 |  | 0.49 | 0.20–1.19 | 0.116 |
| Trp Q4 | 0.66 | 0.37–1.16 | 0.151 |  | 0.98 | 0.54–1.78 | 0.937 |  | 1.01 | 0.48–2.12 | 0.986 |  | 0.69 | 0.24–2.01 | 0.501 |
| Val Q1 | Ref. |  |  |  | Ref. |  |  |  | Ref. |  |  |  | Ref. |  |  |
| Val Q2 | 0.73 | 0.42–1.28 | 0.277 |  | 0.73 | 0.41–1.32 | 0.303 |  | 0.75 | 0.39–1.41 | 0.369 |  | 0.66 | 0.34–1.30 | 0.230 |
| Val Q3 | 0.46 | 0.27–0.79 | 0.005 |  | 0.49 | 0.26–0.90 | 0.022 |  | 0.49 | 0.25–0.98 | 0.043 |  | 0.41 | 0.17–0.89 | 0.045 |
| Val Q4 | 0.75 | 0.43–1.33 | 0.327 |  | 1.09 | 0.59–2.00 | 0.784 |  | 1.12 | 0.53–2.37 | 0.764 |  | 0.83 | 0.25–2.73 | 0.764 |
| His Q1 | Ref. |  |  |  | Ref. |  |  |  | Ref. |  |  |  | Ref. |  |  |
| His Q2 | 1.00 | 0.59–1.70 | 0.987 |  | 1.10 | 0.62–1.95 | 0.747 |  | 1.13 | 0.63–2.05 | 0.677 |  | 1.15 | 0.59–2.24 | 0.672 |
| His Q3 | 0.49 | 0.25–0.94 | 0.033 |  | 0.60 | 0.28–1.30 | 0.198 |  | 0.64 | 0.29–1.39 | 0.256 |  | 0.65 | 0.26–1.62 | 0.357 |
| His Q4 | 0.93 | 0.54–1.58 | 0.779 |  | 1.24 | 0.71–2.16 | 0.454 |  | 1.33 | 0.70–2.54 | 0.379 |  | 1.39 | 0.56–3.50 | 0.479 |
| Arg Q1 | Ref. |  |  |  | Ref. |  |  |  | Ref. |  |  |  | Ref. |  |  |
| Arg Q2 | 0.67 | 0.39–1.15 | 0.142 |  | 0.65 | 0.38–1.12 | 0.124 |  | 0.68 | 0.38–1.19 | 0.174 |  | 0.62 | 0.33–1.15 | 0.132 |
| Arg Q3 | 0.55 | 0.30–1.01 | 0.052 |  | 0.70 | 0.34–1.44 | 0.334 |  | 0.75 | 0.36–1.57 | 0.441 |  | 0.64 | 0.25–1.68 | 0.367 |
| Arg Q4 | 0.75 | 0.43–1.28 | 0.290 |  | 1.01 | 0.58–1.78 | 0.959 |  | 1.12 | 0.55–2.27 | 0.763 |  | 0.89 | 0.33–2.40 | 0.811 |
| Ala Q1 | Ref. |  |  |  | Ref. |  |  |  | Ref. |  |  |  | Ref. |  |  |
| Ala Q2 | 0.68 | 0.39–1.18 | 0.169 |  | 0.75 | 0.43–1.31 | 0.315 |  | 0.79 | 0.43–1.44 | 0.436 |  | 0.76 | 0.39–1.47 | 0.408 |
| Ala Q3 | 0.57 | 0.30–1.09 | 0.088 |  | 0.72 | 0.33–1.55 | 0.397 |  | 0.77 | 0.35–1.70 | 0.514 |  | 0.72 | 0.26–1.99 | 0.528 |
| Ala Q4 | 0.77 | 0.46–1.29 | 0.325 |  | 1.07 | 0.63–1.84 | 0.794 |  | 1.20 | 0.61–2.33 | 0.599 |  | 1.09 | 0.41–2.88 | 0.866 |
| Asp Q1 | Ref. |  |  |  | Ref. |  |  |  | Ref. |  |  |  | Ref. |  |  |
| Asp Q2 | 0.66 | 0.38–1.14 | 0.136 |  | 0.70 | 0.39–1.24 | 0.222 |  | 0.71 | 0.39–1.29 | 0.257 |  | 0.61 | 0.31–1.18 | 0.140 |
| Asp Q3 | 0.54 | 0.30–0.97 | 0.039 |  | 0.69 | 0.34–1.40 | 0.308 |  | 0.70 | 0.34–1.46 | 0.346 |  | 0.54 | 0.21–1.40 | 0.205 |
| Asp Q4 | 0.64 | 0.37–1.11 | 0.110 |  | 0.90 | 0.50–1.63 | 0.732 |  | 0.92 | 0.45–1.90 | 0.831 |  | 0.62 | 0.21–1.81 | 0.379 |
| Glu Q1 | Ref. |  |  |  | Ref. |  |  |  | Ref. |  |  |  | Ref. |  |  |
| Glu Q2 | 0.95 | 0.56–1.62 | 0.848 |  | 0.91 | 0.52–1.59 | 0.747 |  | 0.94 | 0.51–1.72 | 0.835 |  | 0.81 | 0.44–1.48 | 0.488 |
| Glu Q3 | 0.57 | 0.31–1.03 | 0.063 |  | 0.72 | 0.38–1.37 | 0.322 |  | 0.75 | 0.37–1.49 | 0.409 |  | 0.59 | 0.26–1.36 | 0.218 |
| Glu Q4 | 0.75 | 0.42–1.34 | 0.334 |  | 0.90 | 0.50–1.63 | 0.7362 |  | 0.95 | 0.45–2.02 | 0.899 |  | 0.68 | 0.26–1.74 | 0.420 |
| Gly Q1 | Ref. |  |  |  | Ref. |  |  |  | Ref. |  |  |  | Ref. |  |  |
| Gly Q2 | 0.77 | 0.45–1.33 | 0.352 |  | 0.90 | 0.51–1.59 | 0.713 |  | 0.94 | 0.51–1.73 | 0.838 |  | 0.85 | 0.45–1.64 | 0.637 |
| Gly Q3 | 0.58 | 0.32–1.05 | 0.074 |  | 0.80 | 0.41–1.57 | 0.517 |  | 0.85 | 0.42–1.70 | 0.644 |  | 0.74 | 0.30–1.81 | 0.511 |
| Gly Q4 | 0.75 | 0.43–1.31 | 0.305 |  | 0.99 | 0.54–1.81 | 0.976 |  | 1.09 | 0.53–2.22 | 0.824 |  | 0.88 | 0.34–2.32 | 0.800 |
| Pro Q1 | Ref. |  |  |  | Ref. |  |  |  |  |  |  |  | Ref. |  |  |
| Pro Q2 | 0.68 | 0.39–1.19 | 0.176 |  | 0.64 | 0.36–1.14 | 0.128 |  | 0.63 | 0.35–1.13 | 0.122 |  | 0.55 | 0.31–1.00 | 0.049 |
| Pro Q3 | 0.56 | 0.33–0.95 | 0.032 |  | 0.65 | 0.38–1.11 | 0.111 |  | 0.63 | 0.34–1.15 | 0.129 |  | 0.49 | 0.25–0.95 | 0.035 |
| Pro Q4 | 0.60 | 0.33–1.07 | 0.085 |  | 0.73 | 0.39–1.35 | 0.317 |  | 0.70 | 0.34–1.43 | 0.324 |  | 0.49 | 0.21–1.13 | 0.095 |
| Ser Q1 | Ref. |  |  |  | Ref. |  |  |  | Ref. |  |  |  | Ref. |  |  |
| Ser Q2 | 0.58 | 0.34–1.00 | 0.050 |  | 0.65 | 0.37–1.16 | 0.145 |  | 0.63 | 0.33–1.17 | 0.145 |  | 0.51 | 0.26–1.00 | 0.052 |
| Ser Q3 | 0.41 | 0.23–0.74 | 0.003 |  | 0.46 | 0.23–0.92 | 0.027 |  | 0.43 | 0.20–0.92 | 0.030 |  | 0.30 | 0.11–0.79 | 0.015 |
| Ser Q4 | 0.69 | 0.40–1.19 | 0.182 |  | 0.98 | 0.54–1.77 | 0.944 |  | 0.90 | 0.43–1.89 | 0.776 |  | 0.53 | 0.15–1.84 | 0.318 |
| Hyp Q1 | Ref. |  |  |  | Ref. |  |  |  | Ref. |  |  |  | Ref. |  |  |
| Hyp Q2 | 0.99 | 0.59–1.65 | 0.962 |  | 1.11 | 0.63–1.95 | 0.722 |  | 1.12 | 0.63–1.98 | 0.703 |  | 1.13 | 0.62–2.05 | 0.686 |
| Hyp Q3 | 0.63 | 0.34–1.16 | 0.136 |  | 0.84 | 0.41–1.74 | 0.646 |  | 0.87 | 0.42–1.83 | 0.718 |  | 0.88 | 0.40–1.94 | 0.750 |
| Hyp Q4 | 1.19 | 0.67–2.12 | 0.546 |  | 1.33 | 0.71–2.50 | 0.380 |  | 1.42 | 0.72–2.79 | 0.3148 |  | 1.41 | 0.65–3.06 | 0.388 |

*ORs and 95% CIs were estimated using the generalized estimating equations.

Model 1: adjusted for sex, age (60-69 y/70-79 y/≥80 y), BMI (kg/m²), MMSE (score), CES-D (score), education (0-7 y/8-15 y/≥16 y), smoking status (current/not), employment status (yes/no), using of hypnotics, sedatives, or anxiolytics (yes/no), physical activity (MET-min/d), history of stroke, hypertension, ischemic heart disease, dyslipidemia, and diabetes mellitus at baseline, and follow-up period (y).

Model 2: adjusted for energy intake (kcal/d) in addition to the variables in model 1.

Model 3: adjusted for protein intake (g/d) in addition to the variables in model 1.

OR, odds ratio; CI, confidence interval; Ile, Isoleucine; Leu, Leucine; Lys, Lysine; Met, Methionine; Cys, Cystine; Phe, Phenylalanine; Tyr, Tyrosine; Thr, Threonine; Trp, Tryptophan; Val, Valine; His, Histidine; Arg, Arginine; Ala, Alanine; Asp, Aspartic acid; Glu, Glutamic acid; Gly, Glycine; Pro, Proline; Ser, Serine; Hyp, Hydroxyproline; BMI, body mass index; MMSE, Mini-Mental State Examination; CES-D, The Center for Epidemiologic Studies Depression Scale; MET, metabolic equivalents.
