# Supplementary material for: Dietary amino acid intake and sleep duration are additively involved in future cognitive decline in Japanese adults aged 60 years or over: a community-based longitudinal study
Source: BMC Geriatr. 2023 Oct 11;23:653. doi: 10.1186/s12877-023-04359-2 (PMC10568860; doi:10.1186/s12877-023-04359-2)
Supplement: Supplementary file 4 — Supplementary Material 4 [file 12877_2023_4359_MOESM4_ESM.docx]

**Additional File 4**

**Supplemental Table 4**

**File format:** Microsoft word (.docx)

**Title of data:** Multivariable-adjusted longitudinal association between the ratios of amino acid to protein and incidence of cognitive impairment in long-sleepers

**Description of data:** Supplemental Table 4 shows the results of a supplemental analysis. This analysis was performed to interpret the study results from multiple perspectives, although different from the research aim.

**Supplemental Table 4.**

**Multivariable-adjusted longitudinal association between the amino acid ratios to protein and incidence of cognitive impairment in long-sleepers**

|  | Crude* | | |  | Model 1* | | |  | Model 2* | | |
| --- | --- | --- | --- | --- | --- | --- | --- | --- | --- | --- | --- |
|  | OR | 95 %CI | P value |  | OR | 95 %CI | P value |  | OR | 95 %CI | P value |
| Isoleucine, % | 1.04 | 0.64–1.72 | 0.865 |  | 0.92 | 0.56–1.52 | 0.741 |  | 0.92 | 0.56–1.50 | 0.727 |
| Leucine, % | 1.15 | 0.72–1.85 | 0.563 |  | 1.07 | 0.66–1.71 | 0.791 |  | 1.06 | 0.66–1.70 | 0.806 |
| Lysine, % | 1.26 | 0.80–1.98 | 0.318 |  | 1.12 | 0.69–1.82 | 0.653 |  | 1.12 | 0.68–1.82 | 0.661 |
| Methionine, % | 1.01 | 0.64–1.59 | 0.959 |  | 0.88 | 0.52–1.49 | 0.643 |  | 0.88 | 0.52–1.49 | 0.643 |
| Cystine, % | 1.37 | 0.87–2.16 | 0.177 |  | 1.33 | 0.83–2.11 | 0.235 |  | 1.35 | 0.84–2.16 | 0.212 |
| Phenylalanine, % | 1.39 | 0.87–2.24 | 0.170 |  | 1.19 | 0.72–1.98 | 0.494 |  | 1.20 | 0.72–1.98 | 0.484 |
| Tyrosine, % | 1.01 | 0.63–1.62 | 0.977 |  | 0.89 | 0.56–1.43 | 0.640 |  | 0.89 | 0.56–1.43 | 0.637 |
| Threonine, % | 1.22 | 0.74–1.99 | 0.435 |  | 0.90 | 0.53–1.52 | 0.696 |  | 0.89 | 0.53–1.50 | 0.666 |
| Tryptophan, % | 1.24 | 0.76–2.02 | 0.395 |  | 0.91 | 0.56–1.50 | 0.722 |  | 0.92 | 0.56–1.50 | 0.735 |
| Valine, % | 1.14 | 0.70–1.84 | 0.599 |  | 0.91 | 0.56–1.48 | 0.702 |  | 0.91 | 0.56–1.47 | 0.703 |
| Histidine, % | 1.33 | 0.86–2.03 | 0.197 |  | 1.24 | 0.76–2.04 | 0.396 |  | 1.24 | 0.76–2.03 | 0.389 |
| Arginine, % | 1.53 | 0.95–2.46 | 0.081 |  | 1.31 | 0.81–2.10 | 0.267 |  | 1.29 | 0.80–2.09 | 0.290 |
| Alanine, % | 1.24 | 0.79–1.95 | 0.356 |  | 0.92 | 0.57–1.51 | 0.750 |  | 0.92 | 0.57–1.49 | 0.726 |
| Aspartic acid, % | 1.26 | 0.79–2.00 | 0.328 |  | 0.96 | 0.59–1.56 | 0.863 |  | 0.95 | 0.58–1.54 | 0.832 |
| Glutamic acid, % | 1.16 | 0.74–1.82 | 0.522 |  | 1.07 | 0.66–1.73 | 0.794 |  | 1.10 | 0.67–1.80 | 0.710 |
| Glycine, % | 1.15 | 0.71–1.86 | 0.562 |  | 0.90 | 0.54–1.50 | 0.690 |  | 0.89 | 0.53–1.49 | 0.667 |
| Proline, % | 1.35 | 0.86–2.13 | 0.191 |  | 1.22 | 0.76–1.98 | 0.410 |  | 1.25 | 0.77–2.05 | 0.368 |
| Serine, % | 1.32 | 0.83–2.09 | 0.237 |  | 1.06 | 0.65–1.71 | 0.823 |  | 1.06 | 0.66–1.72 | 0.812 |
| Hydroxyproline, % | 1.03 | 0.66–1.61 | 0.884 |  | 0.91 | 0.55–1.51 | 0.727 |  | 0.90 | 0.54–1.50 | 0.688 |

*ORs and 95% CIs were estimated using the generalized estimating equations.

Model 1: adjusted for sex, age (60-69 y/70-79 y/≥80 y), BMI (kg/m²), MMSE (score), CES-D (score), education (0-7 y/8-15 y/≥16 y), smoking status (current/not), employment status (yes/no), using of hypnotics, sedatives, or anxiolytics (yes/no), physical activity (MET-min/d), history of stroke, hypertension, ischemic heart disease, dyslipidemia, and diabetes mellitus at baseline, and follow-up period (y).

Model 2: adjusted for energy intake (kcal/d) in addition to the variables in model 1.
